# Correction: Mapping career patterns in research: A sequence analysis of career histories of ERC applicants

**DOI:** 10.1371/journal.pone.0253832

**Published:** 2021-06-22

**Authors:** The PLOS ONE Staff

There is an error in the order of author affiliations. The publisher apologizes for the error. The correct order is:

Claartje J. Vinkenburg^2^*, Sara Connolly^3¶^, Stefan Fuchs^1¶^, Channah Herschberg^4¶^, Brigitte Schels^1,5¶^

**1** Institute for Employment Research, IAB, Nuremberg, Germany, **2** Independent expert, Amsterdam, the Netherlands, affiliated with VU Amsterdam, **3** Norwich Business School, University of East Anglia, Norwich, United Kingdom, **4** Institute for Management Research, Radboud University, Nijmegen, the Netherlands, **5** Friedrich-Alexander University of Erlangen-Nuremberg, Nuremberg, Germany

* Corresponding author

^¶^ Corresponding author is principal investigator, all other authors appear in alphabetical order, and made equal contributions to data collection, data analysis, and writing
