# Supplementary material for: Mapping career patterns in research: A sequence analysis of career histories of ERC applicants
Source: PLoS One. 2020 Jul 29;15(7):e0236252. doi: 10.1371/journal.pone.0236252 (PMC7390397; doi:10.1371/journal.pone.0236252)
Supplement: S1 File — (DOCX) [file pone.0236252.s001.docx]

**Supplementary Materials LT1.**

**Literature Review on Career Patterns in Research**

We searched several electronic databases for articles published in English that had both “career” and “patterns” or “career patterns” in their title, abstracts, or keywords, as well as “research”, “science” or “academia/academe/academy”. As an alternative for “career patterns”, we searched for articles on “career histories”, “career paths”, “career trajectories”, and “mobility patterns”. This search generated over 130 articles. In a next step, we excluded those articles that were based on career patterns of other samples than researchers, scientists, or academics, but included articles covering samples of PhD-holders leaving academia or of researchers in industry, which meant there were about 67 articles left. From these sources, we concentrated on those published in peer-reviewed scientific journals and most recent books, leaving about 48. Next, we selected articles that were based on or explicitly referred to primary empirical data, thus excluding theoretical conceptualizations of career patterns or review articles. In addition, we searched via the reference lists of all sources thus collected. This helped to add a few more relevant articles. As only three studies out of these empirically distinguish actual career patterns, our final review also includes studies that address career implications of single mobility events (e.g., move from one institution to another, move from industry to academia, or leaving academia). Our search and selection exercise generated a final selection of 40 unique sources published in or before September 2019. These 40 sources, methodologies used, and research career patterns identified, if any, are summarized in Table LT1.

Note: an earlier version of this review based on 23 sources published before 2014 has been published in: Dlouhy K, Vinkenburg CJ, Biemann T. Career patterns. In: Gunz HP, Lazarova M, Mayrhofer W, editors. The Routledge Companion to Career Studies. Abingdon: Routledge; 2019. pp. 242–255. doi:10.4324/9781315674704-15.

***LT.1 Summary of evidence from literature review of research career patterns (40 studies)***

| Author/year | Career patterns identified (if any) | Sample size and nature, study design, and analytical strategy |
| --- | --- | --- |
| Abramo et al 2015 [1] | No classification or typology of career patterns (sequences) in sciences; analysis of the determinants of career advancement (scientific merit; nepotism; social proximity between the candidate and members of the selection committee; geography). Evidence that years of service and co-authored research with members of the committee (particularly the Chair) are key factors in determining success. | Italian data, N=1979 assistant professors, 2008. Cross section data supplemented with bibliometrics. Descriptive statistics, logistic regression analysis of the probability of success. |
| Agarwal & Ohyama 2013 [2] | No classification or typology of career patterns (sequences) in sciences; analysis of the factors influencing career choices (basic/applied scientist; industry/academia) and the respective earnings profiles over years of employment experience. Four types of career and earning trajectories (basic or applied research, industry or academia), sorted by ability and taste for (non)monetary returns. | USA data, N=33,776 doctorate recipients, Survey of Doctorate Recipients (SDR) between 1995 and 2006. Longitudinal data. Probit model, logit and regression analyses of human capital variables, position at time of survey and earnings. |
| Azoulay et al. 2017 [3] | No classification or typology of career patterns (sequences) in sciences; analysis of the determinants of mobility of elite academics, regional mobility increases with research productivity, higher quality of peer environment at distant institutions in relation to home institution, children at high-school age reduce regional mobility | USA data, N=10,051 elite life scientists, reconstruction of their careers (e.g. through CVs, Google searches etc.). Discrete-time hazard rate models for regional mobility events |
| Biemann & Datta 2014 [4] | Typology of career patterns of deans at business schools; based on the definition of four different career positions (member of university research or teaching faculty, administrative position at university, positions in government or industry/company positions) five career types were identified: administrative career, corporate career, research career, government career, and corporate to academic career. Research career is the most frequent pattern | USA data, N=149 deans of accredited business schools in 2006. Optimal matching analysis (OMA) and cluster analysis |
| Bilimoria et al. 2013 [5] | No classification or typology of career patterns (sequences) in sciences; description of differences in the work experiences of men and women at various career stages. Gender differences in career courses, significant differences in terms of work hours, productivity, job satisfaction and compensation through all career stages, women participate in fewer academic career and network development activities in the early and middle career stages and invest more time in teaching than men but less time in research | USA data, N=13,002 full-time faculty members working at four-year institutions, data from 2004 National Study of Postsecondary Faculty, T-tests. |
| Browning et al. 2017 [6] | No classification or typology of career patterns (sequences) in sciences; description of the timing of career trajectories from PhD to research leaders. There are no gender differences in the career progression. Interviewed research leaders report on important career factors such as recognition, mentoring, and early grant success. | Australian data, N=30 senior research leaders at universities. Qualitative biographical interviews and use of information from CVs. |
| Cable & Murray 1999 [7] | No classification or typology of career patterns (sequences) in sciences; analysis of number of job offers, prestige of job offers and salary. Number and prestige of job offers and salary is based on contest mobility (performance) rather than sponsored mobility (prestige of PhD department) | N=159 job seekers using placement services at academic conference in Canada. Survey after one year. Regression analysis. |
| Cañibano et al. 2016 [8] | No classification or typology of career patterns (sequences) in sciences; investigating international mobility rates by gender and research field. International mobility is measured using frequency and duration of research visits. Although women are more internationally mobile, they find that research visits are less frequent, shorter, occur at earlier stages in their career and the visits are to institutions that are closer to their home institution. | Spanish data, N=10,349 researchers with doctorates in 9 research fields. Cross-sectional data, Scientific Information System of Andalusia (SICA), 30 May 2009. Data collected from standardised CVs that researchers are required to upload onto the database. Descriptive statistics and significance tests (p-test, t-test, Mann Whitney) for differences in number, duration and timing of temporary research visits by gender and research area. |
| Carrigan et al. 2017 [9] | No classification or typology of career patterns (sequences) in sciences; investigation of “on-ramping”-strategy , identification of four phases of on-ramping: (1) evaluating the value of transferable non-academic career skills; (2) bridging gaps in experience; (3) coping with both past and present gender discrimination; (4) affirming the value of producing scientific knowledge in academia. | USA data, Semi-structured interviews of N=10 female PhDs who on-ramped (returned) into academia (STEM fields) after workshop participating. Axial coding of the qualitative data. |
| Chubin et al. 1981 [10] | No classification or typology of career patterns (sequences) in sciences; analysis of the impact of early career success (productivity, mentors,...) on later publication record and salary. No explicit career patterns, but evidence for early publications rate predicting later publication rate and salary in academics, not found for non-academic careers | USA data, N=645 doctorates in six disciplines attaining the PhD 1969-1970 from USA universities, survey. Regression analysis. |
| Conley 2005 [11] | No classification or typology of career patterns (sequences) in sciences; description of job positions, tenure status, academic and employment characteristics, institution among men and women in various career stages. Similar career paths of women and men, particularly considering the job positions among younger women and men. | USA data, N=17,600, 1999 National Study of Postsecondary Faculty (NSOPF: 99), survey including retrospective information. Descriptive statistics for different career stages. |
| Conti & Visentin 2015 [12] | No classification or typology of career patterns (sequences) in sciences; investigation of gender differences in a) the employment chances in academia, public administration, or industry after PhD, b) employment in prestigious universities after postdoc training, and c) appointment to professorship. Gender differences exist after controlling for research output and supervisor characteristics. | European data, N=2,345, PhD graduates from elite universities in Switzerland and Sweden between 1999 and 2009. Data collected from universities and web search. Regression analysis. |
| Cruz-Castro & Sanz Menéndez 2010 [13] | No classification or typology of careers patterns (sequences) in science; examination of the relationship between mobility, scientific performance, and tenure. Early permanent positions more likely in internal organizational job markets. | Spanish data, N=1,583 academic scientists from a database of researchers who got their first permanent position between 1997 and 2004. Combination of mail survey and pre-tenure publication record (Science Citation Index). Regression analysis. |
| Dietz & Bozeman 2005 [14] | No classification or typology of career patterns (sequences) in sciences; effects of job mobility, especially between university and industry, grants, first job position and doctorate on later productivity. Number of years in jobs in industry is negatively correlated with the publication rate and positively correlated with the patent rate, job transformations may increase productivity in publications. | USA data, N=1,200 CVs of research scientists and engineers combined with patent data from the USA Patent and Trademark Office database for each respondent. Descriptive statistics, Tobit models, and Poisson models. |
| Dowd & Kaplan 2005 [15] | No classification or typology of careers patterns (sequences) in science; typology of “boundaried” and “boundaryless” tenure-track career experiences, profiles of careers along identity, motivation, tenure concerns, other concerns and career management. Four academic career types based on the parameters of academic positions and career style: “Probationer”, “Maverick”, “Conservationist”, and “Connector”; describe and typify individual careers, not patterns or clusters of careers. | USA data, N=21 tenure-track business professors from three universities, in-depth structured interviews, additional N=13 tenure-track faculty from fourth university used for confirmatory data. Qualitative analysis. |
| Duberley & Cohen 2010 [16] | No classification or typology of career patterns (sequences) in sciences; study on the meaning and availability of career capital among women scientists within the structure of science. Statements about gendered operating of career capital and access to career capital result in curtailed career choices. | UK data, N=31 female academic scientists, in-depth interviews. Qualitative analysis. |
| Duberley et al. 2006 [17] | No classification or typology of career patterns (sequences) in sciences; role of organizational, political, social and cultural contexts for individual career development and management. Institutional contexts for “four scripts which individuals draw on in accounting for their careers” (S. 1139): Organizational careerist, Impassioned scientist, Strategic opportunist, Balance seeker (types of individual careers). | UK and NZ data, N=77 public sector research scientists, interviews. Qualitative analysis. |
| Fumasoli et al. 2015 [18] | No quantitative analysis, empirical study, summary (final chapter in book of same title) of the central findings. Four main stages in academic careers: doctoral studies, post-doc/junior positions, lower-level senior and higher-level senior positions, slow development of intern structures for careers in universities from chair model to department-based model, increased competition for job positions, finances and symbolic resources, increasing relevance of mentoring and career networks, international mobility is helpful in early careers but not indispensable step in academic careers. | Data from 8 countries (Finland, Rumania, Austria, Poland, Switzerland, Croatia, Germany and Ireland).  N=500 interviews with institutional leaders, junior and senior academics, and higher education professionals (HEPROs). Interviews with a common core of questions focusing on governance, academic careers and forms of professionalization. Qualitative analysis. |
| Gulbrandsen & Thune 2017 [19] | No classification or typology of career patterns (sequences) in sciences; Examination whether academics with academic work experience differ from academics with non-academic work experience regarding their research performance and their interaction with external stakeholders, non-academic work experience positively influences external interaction, no evidence for a negative effect of non-academic work experience on scientific productivity/ research performance. | Norwegian data, N=4,400 academic employees from universities & colleges from all academic fields. Survey. Logistic regressions models. |
| Hadani et al. 2012 [20] | No classification or typology of career patterns (sequences) in sciences; impact of academic network characteristics and merit-based criteria (publications) on subsequently obtained job prestige. Evidence for academic network centrality influencing the likelihood to achieve prestigious job position, no influence on further publications. | USA data, N=602 PhD graduates in management science in 2005 - 2007 from 102 USA PhD-granting institutions and were hired for their first academic positions in 2006 – 2008. OLS regression, Logistic regression models. |
| Jagsi et al. 2011 [21] | No classification or typology of career patterns (sequences) in sciences; description of individual characteristics and career outcomes of male and female academic medicals and its impact on career success (receiving a highly notated grant). Gender differences in academic success of academic medical careers. | USA data, N=589 respondents (211 female) health professional doctorates who obtained career development awards to pursue research (K08 and K23 awards) in 2000-2001, survey. Chi-square test, Two-sample T-tests, Logistic regression models. |
| Kahn 1993 [22] | No classification or typology of career patterns (sequences) in sciences; analysis of career progress of men and women after PhD in terms of years until tenure and full professor. Gender differences in academic career progress are mainly existent during tenure process, first evidence for declining differences in younger cohorts. | USA data, N=608 (179 females, 429 males) 24- to 63-year-old PhDs who received the PhD after 1970, biannual Panel Survey of Doctorate Recipients (SDR). Kaplan-Meier-estimations, Hazard Rates. |
| Lawson & Shibayama 2015 [23] | No classification or typology of career patterns (sequences) in sciences; analysis of the impact of international research visits on promotion. Evidence that international research visits are positively associated with rates and speed of promotion (by one year). | Japanese data, N=370, Professors in bioscience. Cross section data, survey conducted in 2010, sent to all professors in bioscience that had received GiA funding over the period 2006-9. Descriptive statistics and survival analysis of the length of time as Associate Professor before promotion to full Professor. |
| Lee et al. 2010 [24] | No classification or typology of careers patterns (sequences) in science; examination of perceived valuable competencies in different career types after graduation, differentiation between academic/public research careers in comparison to technical positions in manufacturing and employment outside conventional technical occupations. Individuals perception of the value of knowledge and skills from doctoral education differ by career type. | UK data, N=102 science and engineering PhD graduates 1998 and 2001 from one research-based university Survey. Variance analysis. |
| Leslie et al. 1998 [25] | No classification or typology of career patterns (sequences) in sciences; analysis of the factors of young male and female youths first choice of college major. Under-representation of women in science and engineering careers are explained by differences in self-concept, self-efficacy, peer group and commitment developed before college. | USA data, Standard integrative review strategies; Cooperative Institutional Research Program (CIRP) files & National Longitudinal Survey of Youth (NLSY). Logistic regression, Multinomial logistic regression, Ordered logistic regression models. |
| Long et al. 1993 [26] | No classification or typology of career patterns (sequences) in sciences; analysis of characteristics of the PhD job and current job influencing the probability and timing of being promoted to the next academic rank. Gender differences in the likelihood to be promoted on the academic track, quantity of publications is more important than quality of publications, gender differences are attributable to differences in levels of variables affecting promotion, differences in expected timing of promotion to associate professor and negative effects of department prestige on promotion to full professor for women. | USA data, N=556 men who received their PhD in biochemistry between 1956 and 1958 or 1961 and 1963, N=450 women who received their PhD between 1956 und 1967, biographical information until 1981 obtained from directory ‘American Men and Women of Science’ or vitae. Event history analysis. |
| Lutter & Schröder 2016 [27] | No classification or typology of career patterns (sequences) in sciences; examination of the likelihood to achieve permanent professorship in sociology based on individual scientific productivity (different kind of publications and co-authorships) and non-meritocratic factors (network size, prestige of department, awards, international mobility), under control of these factors, women are more likely to get tenure than men. | German data, Analysis of career and publication data from person webpages of 1260 faculty members in sociology faculties, time-variable information on productivity and non-meritocratic factors. Cox regression models. |
| Marini 2017 [28] | No classification or typology of career patterns (sequences) in sciences; examination of the likelihood to achieve promotion through a new habilitation procedure (ASN) based on individual scientific productivity, traditional seniority pattern is still more common than fast promotion. | Italian data, Analysis of secondary data (CVs and publications) of all applicants in the ASN system that applied for full professorships in four disciplines in 2012 (N between 2,253 & 5,269). Logistic regression models. |
| Miller et al. 2005 [29] | No classification or typology of career patterns (sequences) in sciences; study of path-dependency in careers analysing the impact of prestige of previous affiliations and research success on later affiliation prestige and research success (year 1, 6, 11, 16 after PhD) under control of training department prestige, mentor, and gender. Careers through the prestige hierarchy, women are more likely than men to dismount in the prestige hierarchy within the first 16 years of their careers. | USA data, N=298 graduates from 1977 through 1985 from 36 training institutions, biographical information for 16 years after graduation taken from National Faculty Directory, the McGraw-Hill-Directory of Management Faculty, the Academy of Management on-line directory and the Social Sciences Citation Index including web search and person networks. Structural equation model. |
| Nerad & Cerny 1999 [30] | No classification or typology of career patterns (sequences) in sciences; description of men and women in postdoc and no postdoc positions by postdoc history, age at tenure, family and postdoc appointments. Postdoctoral appointments vary in its relevance and function for the career between disciplines (mathematics and biochemistry) and gender. | USA data, N=3,667 PhD recipients in six fields at 61 universities from 1982 to 1985, PhDs – Ten Years Later survey. Descriptive analysis. |
| Ooms et al. 2018 [31] | No classification or typology of career patterns (sequences) in sciences; Examination of effects of heterogeneity on career advancement along a prototypical academic career path (PhD to full professor). Heterogeneity in research orientation is helpful for career advancement, heterogeneity stemming from gender hamper career advancement. Heterogeneity between mentor and researcher is useful in early careers, but hinders advancement later on. | Data from Germany and the Netherlands, N=248 academics at two leading European universities of technology. Web-based survey. Multinomial logistic regression, Sequential logistic regression models. |
| Pezzoni et al. 2012 [32] | No classification or typology of career patterns (sequences) in sciences; Examination of effects of social capital on promotion to professorial positions. Influence of individual characteristics, productivity score (publications), institutional affiliations and collaborations (scientific human capital) and networks to professors within discipline (political capital); Political capital is particularly important in Italy, scientific human capital particularly important in France. | Italian and French data, academic physicists observed between 2000 and 2003/2005 (n=813 for France, n=469 for Italy) obtained from the Ministries of Education. Logistic regression models. |
| Romanin & Over 1993 [33] | No classification or typology of career patterns (sequences) in sciences; description of men and women at lecture level or above in terms of career development (age at honours, at PhD, at first tenured job), career paths (graduate from present university, regional mobility, full-time tutor, career breaks), performance (publications, conferences, research time), ... and family commitments. Evidence for gender-differences in parameters regional mobility and commitment for housework, no differences in self-rated performance also after controlling for number and age of children. | Australian data, N=309 women and men in arts or sciences at universities sampled from 1988 Commonwealth Universities Yearbook and university calendars und handbooks, survey. Variance analysis. |
| Rosenfeld & Jones 1987 [34] | No classification or typology of career patterns (sequences) in sciences; analysis of regional job mobility of men and women and the interplay between regional mobility and job characteristics  Gender differences in regional job mobility, mobility for the first job increases probability to be on tenure track and influences later achievements, “men’s mobility is more affected than women’s by their life-cycle stage and women’s very slightly more by their career position” (p. 510) | USA data, N=311 women and 311 men sampled from the 1981 Directory of the American Psychological Association (APA) who have received their PhD between 1965 and 1974 and whose first job after PhD was in an academic institute, biographical data from the directory were completed with additional data from 1970 Census population, 1962-82 National Unit Book Catalogue and Social Science und Science Citation Index of 1962 to 1981. Logistic regression, Event history analysis. |
| Sağlamer et al 2018 [35] | No classification or typology of career patterns (sequences) in sciences; gender differences in academic career development, results indicate different obstacles for both genders, corresponding to traditional family ideology and gendered division of labour, confirmation of gendered domestic roles and responsibilities, no gender differences in academic performance, with exception long-term mobility. | Turkish data. Data from 7 state universities, quantitative statistical information about the universities and the students. Additional qualitative data through semi-structured group-interviews and questionnaires for N=112 academics. Additional comparative analyses of the representation of women in academia for Turkey, the EU and OECD. |
| Vázquez-Cupeiro & Elston 2006 [36] | No classification or typology of career patterns (sequences) in sciences; study on women’s career constraints coming from university culture and recruitments practices. Recruitment patterns based on internal (departmental) networks excluding women, | Spanish data, N=33 lecturers and professors (16 female, 17 male) working in academic departments of psychology and engineering in three Madrid universities. Qualitative study with open-ended, semi-structured, in-depth interviews. |
| Wessel & Keim 1994 [37] | Classification of career ladders towards university presidency based on the experience of presidents in diverse job positions (faculty, dept. chair, chair, academic VP), two career ladders leading to the private, four-year American college presidency: Academic Career Pattern and Administrative Career Pattern, identification of variations in both career ladders | USA data, N=270 presidents of colleges and universities in 47 US states in 1991. Interviews, coding. |
| Winchester et al. 2006 [38] | No classification or typology of career patterns (sequences) in sciences; analysis of the existence of barriers in academic promotion process for women considering promotion practices and success rates. Similar application rates and success rates of women and men, some higher rates of women on the professor level, still underrepresentation of women on senior levels, political initiatives supporting participation and success of women through part-time and non-traditional careers. | Australian data, Content analysis of documents from all Australian universities, interviews with gatekeepers from 17 universities, analysis of data on promotions from 16 universities in 2000-2002. Qualitative study. |
| Ylijoki & Henriksson 2017 [39] | Exploration of career-building of academics during early career stage, construction of five dominant career scenarios based on individual perspectives on core commitment, career risk, career support, and stance towards the university: “the Novice of the Academic Elite”, ”the Victim of the Teaching Trap”, “the Academic Worker”, “the Research Group Member” and “the Academic Freelancer”. Boundaries in careers are based on cultural capital. | Finnish data. Narrative approach. Based on 3 focus group discussions with early career academics (N=12) in social sciences. |
| Youtie et al. 2013 [40] | No classification or typology of career patterns (sequences) in sciences; comparative study of scientific recognition by peer scientists in early career stages (6 years after PhD) and mid-career stages (12 years after PhD), fast completion of PhD and record of independent postdoctoral research is important for recognition at early career in the USA only and for recognition at mid-career stages in both countries; further work experience, research leadership, external grant income, prizes from professional associations are predictors for USA researches, but less influential in Europe | Comparative study for the USA and various European countries, N=76 creative researchers in the fields of nanotechnology and human genetics from the USA and Europe, identified through survey nominations and prize winnings were matched with a comparison group (N=76) with similar early career publishing characteristics. Data collection from CVs and the Web of Science. Probit regression models for early and mid-career phases. |

References supplementary materials (review table on career patterns in research)

1. Abramo G, D’Angelo CA, Rosati F. The determinants of academic career advancement: Evidence from Italy. Sci Public Policy. 2015. doi:10.1093/scipol/scu086

2. Agarwal R, Ohyama A. Industry or Academia, Basic or Applied? Career Choices and Earnings Trajectories of Scientists. Manage Sci. 2013;59: 950–970. doi:10.1287/mnsc.1120.1582

3. Azoulay P, Ganguli I, Graff Zivin J. The mobility of elite life scientists: Professional and personal determinants. Res Policy. 2017;46: 573–590. doi:10.1016/j.respol.2017.01.002

4. Biemann T, Datta DK. Analyzing Sequence Data: Optimal Matching in Management Research. Organ Res Methods. 2014;17: 51–76. doi:10.1177/1094428113499408

5. Bilimoria D, Liang X, Carter SD, Turell JM. Gender differences in the academic work experiences of faculty at early, middle and late career stages. In: Burke RJ, Vinnicombe S, Moore LL, Blake-Beard S, editors. Handbook of Research on Promoting Women’s Careers. Cheltenham: Edward Elgar ; 2013. pp. 304–325. doi:10.4337/9780857938961.00023

6. Browning L, Thompson K, Dawson D. From early career researcher to research leader: survival of the fittest? J High Educ Policy Manag. 2017;39: 361–377. doi:10.1080/1360080X.2017.1330814

7. Cable DM, Murray B. Tournaments versus sponsered mobility as determinants of job search success. Acad Manag Jounal. 1999;42: 439–449.

8. Cañibano C, Fox MF, Javier Otamendi F. Gender and patterns of temporary mobility among researchers. Sci Public Policy. 2016. doi:10.1093/scipol/scv042

9. Carrigan C, O’Leary K, Riskin E, Yen J, O’Donnell M. On-ramping: following women scientists and engineers through their transition from nonacademic to faculty careers. J Technol Transf. 2017;42: 98–115. doi:10.1007/s10961-015-9460-5

10. Chubin DE, Porter AL, Boeckmann ME. Career Patterns of Scientists: A Case for Complementary Data. Am Sociol Rev. 1981;46: 488–496. doi:10.2307/2095269

11. Conley VM. Career paths for women faculty: Evidence from NSOPF:99. New Dir High Educ. 2005;130: 25–39.

12. Conti A, Visentin F. Science and engineering Ph.D. students’ career outcomes, by gender. PLoS One. 2015;10. doi:10.1371/journal.pone.0133177

13. Cruz-Castro L, Sanz-Menéndez L. Mobility versus job stability: Assessing tenure and productivity outcomes. Res Policy. 2010;39: 27–38. doi:10.1016/j.respol.2009.11.008

14. Dietz JS, Bozeman B. Academic careers, patents, and productivity: industry experience as scientific and technical human capital. Res Policy. 2005;34: 349–367. doi:10.1016/j.respol.2005.01.008

15. Dowd KO, Kaplan DM. The career life of academics: Boundaried or boundaryless? Hum Relations. 2005;58: 699–721.

16. Duberley J, Cohen L. Gendering career capital: An investigation of scientific careers. J Vocat Behav. 2010;76: 187–197.

17. Duberley J, Cohen L, Mallon M. Constructing scientific careers: Change, continuity and context. Organ Stud. 2006;27: 1131–1151. doi:10.1177/0170840606064105

18. Fumasoli T, Goastellec G, Kehm BM. Academic Careers and Work in Europe: Trends, Challenges, Perspectives. In: Fumasoli T, Goastellec G, Kehm MB, editors. Academic Work and Careers in Europe: Trends, Challenges, Perspectives ESF EUROAC project. Cham: Springer International Publishing; 2015. pp. 201–214. doi:10.1007/978-3-319-10720-2_10

19. Gulbrandsen M, Thune T. The effects of non-academic work experience on external interaction and research performance. J Technol Transf. 2017;42: 795–813. doi:10.1007/s10961-017-9556-1

20. Hadani M, Coombes S, Das D, Jalajas D. Finding a good job: Academic network centrality and early occupational outcomes in management academia. J Organ Behav. 2012;33: 723–739. doi:10.1002/job.788

21. Jagsi R, DeCastro R, Griffith KA, Rangarajan S, Churchill C, Stewart A, et al. Similarities and Differences in the Career Trajectories of Male and Female Career Development Award Recipients. Acad Med. 2011;86: 1415–1421. doi:10.1097/ACM.0b013e3182305aa6

22. Kahn S. Gender Differences in Academic Career Paths of Economists. Am Econ Rev. 1993;83: 52–56. doi:10.2307/2117639

23. Lawson C, Shibayama S. International research visits and careers: An analysis of bioscience academics in Japan. Sci Public Policy. 2015;42: 690–710. doi:10.1093/scipol/scu084

24. Lee H, Miozzo M, Laredo P. Career patterns and competences of PhDs in science and engineering in the knowledge economy: The case of graduates from a UK research-based university. Res Policy. 2010;39: 869–881. doi:10.1016/j.respol.2010.05.001

25. Leslie LL, McClure GT, Oaxaca RL. Women and Minorities in Science and Engineering: A Life Sequence Analysis. J Higher Educ. 1998;69: 239–276. doi:10.2307/2649188

26. Long JS, Allison PD, McGinnis R. Rank Advancement in Academic Careers: Sex Differences and the Effects of Productivity. Am Sociol Rev. 1993;58: 703–722. doi:10.2307/2096282

27. Lutter M, Schröder M. Who becomes a tenured professor, and why? Panel data evidence from German sociology, 1980–2013. Res Policy. 2016;45: 999–1013. doi:10.1016/j.respol.2016.01.019

28. Marini G. New promotion patterns in Italian universities: Less seniority and more productivity? Data from ASN. High Educ. 2017;73: 189–205. doi:10.1007/s10734-016-0008-x

29. Miller CC, Glick WH, Cardinal LB. The allocation of prestigious positions in organizational science: accumulative advantage, sponsored mobility, and contest mobility. J Organ Behav. 2005;26: 489–516. doi:10.1002/job.325

30. Nerad M, Cerny J. Postdoctoral Patterns, Career Advancement, and Problems. Science (80- ). 1999;285: 1533–1535. Available: http://www.jstor.org/stable/2898106

31. Ooms W, Werker C, Hopp C. Moving up the ladder: heterogeneity influencing academic careers through research orientation, gender, and mentors. Stud High Educ. 2018; 1–22. doi:10.1080/03075079.2018.1434617

32. Pezzoni M, Sterzi V, Lissoni F. Career progress in centralized academic systems: Social capital and institutions in France and Italy. Res Policy. 2012;41: 704–719. doi:10.1016/j.respol.2011.12.009

33. Romanin S, Over R. Australian academics: Career patterns, work roles, and family life-cycle commitments of men and women. High Educ. 1993;26: 411–429. doi:10.1007/BF01383736

34. Rosenfeld RA, Jones JA. Patterns and Effects of Geographic Mobility for Academic Women and Men. J Higher Educ. 1987;58: 493–515. doi:10.2307/1981784

35. Sağlamer G, Tan MG, Çebi PD, Çağlayan H, Gümüşoğlu NK, Poyraz B, et al. Gendered patterns of higher education in Turkey: Advances and challenges. Womens Stud Int Forum. 2018;66: 33–47. doi:10.1016/j.wsif.2017.11.002

36. Vázquez-Cupeiro S, Elston MA. Gender and academic career trajectories in Spain: From gendered passion to consecration in a Sistema Endogámico? Empl Relations. 2006;28: 588–603. doi:10.1108/01425450610704515

37. Wessel RD, Keim MC. Career Patterns of Private Four-Year College and University Presidents in the United States. J Higher Educ. 1994;65: 211–225.

38. Winchester H, Lorenzo S, Browning L. Academic women’s promotions in Australian universities. Empl Relations. 2006;28: 505–522.

39. Ylijoki O-H, Henriksson L. Tribal, proletarian and entrepreneurial career stories: junior academics as a case in point. Stud High Educ. 2017;42: 1292–1308. doi:10.1080/03075079.2015.1092129

40. Youtie J, Rogers J, Heinze T, Shapira P, Tang L. Career-based influences on scientific recognition in the United States and Europe: Longitudinal evidence from curriculum vitae data. Res Policy. 2013;42: 1341–1355. doi:10.1016/j.respol.2013.05.002
