# Supplementary material for: Mapping career patterns in research: A sequence analysis of career histories of ERC applicants
Source: PLoS One. 2020 Jul 29;15(7):e0236252. doi: 10.1371/journal.pone.0236252 (PMC7390397; doi:10.1371/journal.pone.0236252)
Supplement: S2 File — (PDF) [file pone.0236252.s002.pdf]

Supplementary Materials: Survey questions

a) StG survey

Welcome to the online ERCAREER inventory. Thank you very much for your interest in this research. We kindly remind you that you have previously provided consent for use of your application data for evaluative purposes to the ERC executive agency, which is why you are included in this project. ERCAREER's objective is to formulate specific and concrete recommendations for the development and improvement of the ERC's evidence-based policies, procedures, and practices to achieve greater gender balance among ERC applicants and grantees in particular, thereby contributing to the strive for gender equality in the European Research Area in general. If you have any questions or require more information about this project, please contact the researchers at: project.ercareer@gmail.com

Additional notes

- Please use the arrow buttons to navigate through the inventory.
- Some questions are obligatory, in order to ensure the appropriate flow of the inventory and the most optimal results for our analyses.
- If you interrupt or have to stop the inventory and would like to continue at a later stage, simply close the screen. You can enter the inventory and resume at the same question at a later time by following the link in your email.

Section 1 - Your PhD (or equivalent doctoral degree)

We would like to start by asking a few questions about your life and career before and during your PhD studies (or equivalent).

Q1 Which institution awarded your PhD (or equivalent)? Please write the full name of the institution/university.
Open text

Q2 In which country was the institution located?
[Dropdown menu] Countries

Q3 Did you have any children (biological/adopted/step children) before or during your PhD?
Options: Yes, No

If No Is Selected, Then Skip To Q6

Q4 How many children did you have before or during your PhD?
Options: 1-6

Q5 Can you please give the date(s) of birth?
[Dropdown menu] MM/YYYY

Q6 Did you have work experience of more than 6 months prior to your PhD?

Options: Yes, No

If Yes Is Selected for Q6, If not skip to Q9

Q7 To what extent did this previous work experience help your PhD research?
Options: Not at all, A limited extent, Some extent, A significant extent, It was essential, Not applicable

Q8 To what extent did this previous work experience help your post-doctoral career?
Options: Not at all, A limited extent, Some extent, A significant extent, It was essential, Not applicable

Q9 Since completing your PhD (or equivalent) have you changed your field of research? For example, a move from one subdomain/subpanel of the ERC to another would signify a different field.
Options: No, Yes and please specify the date(s) you changed field. MM/YYYY

Q10 Did you become an independent researcher at some point during your career? (undertaking research or leading a research group without being supervised)
Options: Yes, No

If No Is Selected, Then Skip To Q12

Q11 Can you please specify the date you became an independent researcher?
[Dropdown menu] MM/YYYY

Section 2 – Professional employment since your PhD (or equivalent doctoral degree)

In this section, we refer to "spells of employment". This refers to a period of employment during which both position and institution stay the same. This may include spells, which started before you were awarded your PhD but finished afterwards. If your contract type or number of hours changed during the spell you can indicate this in the survey. We will be asking you about any visiting or honorary positions or any leave taken within every spell of employment – so if any of these occurred whilst you had a contract these should not be considered as separate spells. We will be asking you about any spells of unemployment or leave taken when you were not employed in Section 4. We ask about 20 questions for every spell of employment after your PhD. The questions will be similar for each spell, which may seem repetitive but is necessary for the purposes of this project.

Q12 How many spells of employment have you held since your PhD (or equivalent)?
[Dropdown menu] 1-20

Please do not include periods of unemployment or leave taken when you were not employed - we will ask about these in Section 4. Please note that after you filled out the questions on your spells of employment, there will be an opportunity to add spells in case you were mistaken in the number filled out below.

Q13 What was the start date of your [nr of position] position?
[Dropdown menu] MM/YYYY

Q14 What was the end date of your [nr of position] position? If you are still working in this position, please select the current month and current year.
[Dropdown menu] MM/YYYY

We use the following classifications of the type of employment: **Post doc** - Research Assistant/Associate, Research Fellow, Researcher, Staff researcher; **Lecturer** - Scientist, Senior Researcher, (Junior) group/team leader, Assistant Professor, Lab head, Research Scientist, Principal Investigator, Staff scientist, Senior post-doctoral research fellow, Senior Research Scientist; **Senior lecturer** - Senior Scientist, Professor of Applied, Collaborative Researcher, Clinician Scientist, Chargé de recherche, Associate Professor, Habilitation, Permanent research scientist, Reader; **Professor** - Research Director, Full Professor, Directeur de recherché; **Other** - Consultant, Engineer, Special Researcher, Research Professor
For each position after your PhD

Q15 Type of employment	Q16 Institution type	Q17 Name of institution
Options: Post doc, Lecturer, Senior lecturer, Professor, Other	*Options:* Higher education/ University, Not for profit research institution, Commercial research organization, Hospital/ Clinic, Government, Private organization, Other	Open text

Q18 In which country was the institution / organization located?
[Dropdown menu] Countries

We would like to learn more about your working hours and type of contract you had during your [nr of position] position after your PhD (or equivalent doctoral degree).

Q19 Contract type	Q20 Contract length	Q21 Contract hours	Q22 Actual hours	Q23 Reason for working less than full-time
Options: Full-time, Part-time	*Options:* Fixed term, Permanent	Open text	Open text	*Options:* Family responsibilities, Further study, Health, Only work available, Pursuing other interests, Quality of life, Research project (externally funded), Other

Q24 Did any of the following change during this spell of employment? [Multiple answers possible]
Options: No, nothing changed; Yes, my contract from full time to part time; Yes, my contract from part time to full time; Yes, my contract from fixed term to permanent; Yes, my contract from permanent to fixed term; Yes, my number of hours: more hours; Yes, my number of hours: less hours;
Yes, other ____________________

Below we would like to learn more about the activities you are/were involved in during your [nr of position] position after your PhD. Please make sure the percentages add up to a 100.

Q25 Main duties	Q26 Time involved in research	Q27 Involved in teaching?	Q28 Time involved in teaching	Q29 Involved in administration or management tasks	Q30 Time involved in administration or management	Q31 Involved in other activities (e.g. clinical responsibilities)	Q32 Time involved in other activities
Options: Research, Not research	%	*Options:* Yes, No	%	*Options:* Yes, No	%	*Options:* Yes, No	%

Q33 How strong is/was the link between the teaching and your research?
Options: Very strong, Strong, Neither strong nor weak, Weak, Very weak

Q34 What other activities were you involved in? ____________________

During this spell of employment

Q35 Was your research interdisciplinary?	Q36 What was the main funding source for your research?
Options: Yes, No	*Options:* Institutional funding program, National government research funding program, International research funding program, No additional funding support apart from contract, Other.

Q37 Did you have other paid appointments at this time? [Multiple answers possible]
Options: No; Yes, part time lecturing position; Yes, part time research position; Yes, part time clinical position; Yes, part time government work; Yes, other ____________________

Q38 Did you have other unpaid/ honorary/ visiting appointments at this time? [Multiple answers possible]
Options: No; Yes, honorary appointment; Yes, visiting appointment;
Yes, other ____________________

Q39 Did you have a spouse or partner during this spell?
Options: Yes, No

If Yes Is Selected for Q39

Q40 Is/was your spouse or partner employed at this time?
Options: No; Yes, in full time employment; Yes, in part time employment, Other

Q41 Did you have any children (biological/adopted/step children) at this time?
Options: Yes, No

If No Is Selected, Then Skip To Q44

Q42 How many children did you have during this spell?
Options: 1-6

Q43 Can you please give the date(s) of birth?
[Dropdown menu] MM/YYYY

Q44 Did you have significant care responsibilities for others during this spell of employment? (e.g., care for children, other [extended] family members or other dependents)
Options: None, Sole, Main (>50% but < 100%), Equal, Shared (>0 but < 50%)

Q45 Were there any other significant events (e.g. ill health [own/family], bereavement, etc)?
Options: No, Yes (please specify) ____________________

Q46 Did you take any leave during this spell of employment? (e.g., research leave, parental leave, military leave, medical training, leave due to illness, etc)
Options: Yes, No

If No Is Selected, Then Skip To Next Spell of Employment

Q47 What kind of leave did you take at this time? [Multiple answers possible]

Options: Research leave, Parental leave (incl. maternity leave, paternity leave), Military leave, Medical training, Leave due to long-term illness, Other

If answered Yes in Q47 to any type of leave.

Q48 How many periods of leave - Military leave, Medical training, Leave due to long-term illness, Other - did you take?
Options: 1-3

Q49 What was the start date of your leave?
[Dropdown menu] MM/YYYY

Q50 What was/will be the end date of your leave?
[Dropdown menu] MM/YYYY

What type of leave did you take at this time?

Q51 Did you take paid or unpaid leave?
Options: Paid, Unpaid

Q52 Did you take full-time or part-time leave?
Options: Full-time, Part-time

Answer If Parental leave Is Selected in Q47

Q53 What kind of parental leave did you take at this time? [Multiple answers possible]
Statutory = minimum legal entitlement
Options: Statutory maternity leave, Optional maternity leave, Statutory paternity leave, Optional paternity leave, General parental leave, Other.

Q54 How many periods of parental leave - Statutory maternity, Optional maternity leave, Statutory paternity leave, Optional paternity leave, General parental leave, Other - leave did you take?
Options: 1-3

Q55 What was the start date of your parental leave?
[Dropdown menu] MM/YYYY

Q56 What was/will be the end date of your Statutory maternity leave?
[Dropdown menu] MM/YYYY

What type of parental leave did you take at this time?

Q57 Did you take paid or unpaid leave?
Options: Paid, Unpaid

Q58 Did you take full-time or part-time leave?
Options: Full-time, Part-time

We would now like to ask about how your parental leave was planned. For each period of parental leave:

Q59 Did your employer discuss other ways of managing your workload instead of taking leave?	Q60 Did you plan your leave with your employer?	Q61 Was your employer supportive of your plans?	Q62 Was your supervisor supportive of your plans?	Q63 Did you discuss your plans with work colleagues?	Q64 Were your work colleagues supportive of your plans?

Options: Yes, No

Q65 Did your employer or supervisor remain in contact during your parental leave(s)?
Options: Yes, regular; Yes, occasional; No

Q66 Did you experience any difficulties in returning to work after your parental leave(s)?
Options: Yes, No

Q67 Please write in any examples of what helped or hindered your return to work after your parental leave(s). _____________

For each new spell of employment

Q68 Did any of the following change between this and your next spell of employment? [Multiple answers possible]
Options: Yes, my role; Yes, my institution; Yes, geographic location within same country; Yes, country; No

Answer if any Yes is selected in Q68

Q69 Why did you decide on this change? [Multiple answers possible]
Options: End of contract, Promotion, Better facilities, Better institution, Better contract, Return to country of birth,
Other ____________________

Q70 If you were married or in a partnership at this time, did this change in employment involve a dual hire by the same employer?
Options: Not applicable, Yes, No

Q71 If you had the choice of moving but did not move, why not? [Multiple answers possible]
Options: Not applicable, Excellence of current institution, Excellence of current research group, Family or other responsibilities, Career of my partner, Other ____________________

To check that all spells of employment have been covered

Q72 You indicated that the start date of this spell of employment was [Month/Year] and that the end of this spell of employment was [Month/Year]. Is this the position you are currently working in?
Options: Yes, No

If Yes to Q72 go to Section 3

Q73 Have you forgotten to mention a spell of employment?
Options: No, Yes, I had another spell of employment (2)

If Yes is selected, routed to completed the full set of questions on this spell.

Section 3 - Career choices made and institutional support

We already know whether or not you have taken career breaks or leave since completing your PhD.

Q74 Have there been (other) times where you have considered but not taken any career breaks or leave?
Options: Yes, No

Answer If Yes Is Selected in Q74

Q75 Why did you decide not to take the career break or leave? __________________

We already know whether or not you have worked part time since completing your PhD.

Q76 Have there been (other) times where you have considered but not requested part time employment?
Options: Yes, No

Answer If Yes Is Selected in Q76

Q77 Why did you decide not to request part time employment? __________________

We would like to know whether there is a formal institutional or national system / mechanism which takes account of "time to care" in promotion or tenure decisions, performance measurement or in research time allocation. [Select all that apply]

Q78 **Extension of window**. Stopping the clock for tenure and promotion decisions (e.g. 12 months per child or actual time of leave taken into account)
Q79 **Adaption of criteria**. Compensation for part-time work (e.g. working 80% or 0.8 FTE implies criteria for promotion of research time allocation are set at 80%)
Q80 **Compensation for time to care** (e.g. temporary reduced teaching load to invest in research productivity upon return)

Options: I am not aware of this support, I am aware that this exists, This is available in my institution, I have used this.

Q81 What do you think are the 'most' important organizational factors in supporting the interface between work and family? [Select all that apply]
Options: Availability of good childcare, Contact/keeping in touch with department while away, Availability of other care support, Flexible working, Guarantee for same job when returning after a career break, Less than full time working initially after break or leave, building up to full time, Mentoring, Peer networks, Shorter working hours, Training/retraining, Supervisor support,
Other ____________________

Were any of the following working conditions influential in your current choice of employer?

Q82 Availability of reduced hours (part time)
Q83 Availability of annualised hours (working fixed number of hours a year, flexibility in when to work)
Q84 Availability of compressed hours (e.g. 4 * 9 contract)
Q85 Availability of flexi-time/ flexible hours
Q86 Availability of term time (during the school year) working
Q87 Availability of another type of flexible contract

Options: Never been important, became important after I had children, always been important

Section 4 - Spells outside of employment

We are now interested in times when you did not have an employment contract.

Q88 Since completing your PhD, have there been times where you have been unemployed or where you have taken full time leave outside of a contract?
Options: Yes, No

If No Is Selected, Then Skip To Q95

Q89 Are you currently out of employment?
Options: Yes, No

Q90 How many spells out of employment did you have since your PhD?
Options: 1-5, More than 5.

Q91 Why have you been unemployed or on full time leave? [Multiple answers possible]
Options: No paid employment available, To cover gap between two known employment spells (e.g., contract ends June, new contract starts September), To travel, start up business, For care reasons (e.g. taking care of children without formal paid / unpaid leave options),
Other ____________________

Q92 What was the start date of your first spell of unemployment or full time leave?
[Dropdown menu]

Q93 What was/will be the end date of your first spell of unemployment or full time leave?
[Dropdown menu]

Q92 and Q93 repeated when multiple spells of unemployment or full time leave are indicated

Q94 What was your source of income during this time/ these times? [Multiple answers possible]
Options: Unemployment benefits, Social security benefits, Savings, Partner's income, None,
Other ____________________

Section 5 - Your life outside of work

Q95 At present, do you have a spouse or partner?
Options: Yes, No

If No Is Selected, Then Skip to Q107

Q96 Since what year have you and your spouse or partner been together? YYYY

Q97 Do you and your spouse or partner live in the same household?
Options: Yes, all of the time, Yes, most of the time, No

Q98 Is your spouse or partner employed?
Options: Yes, in full time employment, Yes, in part time employment, No, in education, No, taking leave, No, full time care responsibilities, No, other ____________________

Q99 What is the highest formal qualification of your spouse or partner?
Options: Doctorate/DPhil/PhD, MBA, Professional Law/ Financial/ Business qualification, Masters degree, Bachelors degree, Secondary education, Other ____________________

Q100 Is your spouse or partner also a scientist/researcher/academic?
Options: Yes, No

Answer If your partner is employed? Otherwise skip to Q107

Q101 Is your partner working in a similar institution?
Options: Yes, No

Q102 Is your spouse or partner working in the same institution?
Options: Yes, No

Q103 How many hours per week does your spouse or partner work?

Q104 How easy has it been to combine dual careers?
Options: Very difficult, Difficult, Neither easy nor difficult, Easy, Very easy

Q105 During your relationship, whose career has been more important?
Options: Mine, Mostly mine, Both equally, Mostly my spouse or partner's career, My spouse or partner's career, Not applicable

Q106 For a successful research career, you need a spouse or partner who takes care of the family and household.
Options: Strongly disagree, Disagree, Neither agree nor disagree, Agree, Strongly agree

Section 6 - Applying to the ERC for StG funding

In this section, we are interested to know more about your StG 2012 ERC application.

Why did you apply for StG funding? I applied for StG funding because of

Q107 the amount of funding
Q108 the duration of funding
Q109 the possibility to set up my own research group
Q110 the opportunity to receive funding for my own position
Q111 its support for basic research
Q112 the possibility to choose my own research priorities
Q113 the grant's positive reputation
Q114 a lack of other funding opportunities.

Options: 1-5
1 = Does not apply at all, 5 = Fully applies

Q115 Have you applied to other funding agencies for support with this research? [Select all that apply]
Options: No, only to the ERC; Yes, to other institutional research funding programs; Yes, to other national research funding programs; Yes, to other international research funding programs;
Yes, other ____________________

Q116 Have you made other applications to the ERC? [Multiple answers possible]
Options: No; Yes, StG 2007; Yes, StG 2009; Yes, StG 2010; Yes, StG 2011; Yes, StG 2013; Yes, CoG 2013; Yes, AdG 2008; Yes, AdG 2009; Yes, AdG 2010; Yes, AdG 2011; Yes, AdG 2012; Yes, AdG 2013; Yes, other ____________________

Q117 In making your most recent ERC application did you receive support from within your institution?
Options: Not at all, Limited, Occasional, Extensive, All

Answer If received support from within institution in making your most recent ERC application

Q118 What type of support did you receive? [Select all that apply]
Options: Formal support, Informal support, Supervisor support, Mentoring support

Q119 In making your most recent ERC application did you receive support from outside your institution?
Options: Not at all, Limited, Occasional, Extensive, All

Q120 Did you have a mentor outside your institution?
Options: Yes, No

Section 7 - The ERC evaluation procedure

From your point of view: How influential were the following aspects on the evaluation of your proposal.

Options: 1 – 5
1 = Not important at all, 5 = Very important

Q121 My number of publications
Q122 The reputation of the journals I have published in
Q123 The reputation of my coauthors
Q124 The awards I have received
Q125 The number of patents I have been granted
Q126 The amount of editorial boards and committee memberships
Q127 The reputation of my (preferred) host institution
Q128 My project presentation at ERC
Q129 The quality of my proposal
Q130 My previous work in the research field

Please rate the following aspects of the StG evaluation procedure.

Options: 1 – 5
1 = Very poor, 5 = Excellent

Q131 The swiftness (speed) of the evaluation process
Q132 Information given on the status of the evaluation process status
Q133 Quality of the documentation provided by the ERC
Q134 The composition of the review panel (Interview)
Q135 The quality of the evaluation report on my proposal
Q136 The process of the contract negotiations with the ERC after funding decision
Q137 The overall setup of the evaluation procedure

Section 8 - Your career aspirations

Q138 What are your long-term career goals? i.e., Where do you want to be in 10 years time?
Options: Tenured, full professor, Leading research position, Leading management position, Leading clinical position, Group leader or lab head, I don't know,
Other ____________________

Q139 In the long-run what sort of institution do you want to work in?
Options: Higher education/ University, Not for profit research organization, Commercial research organization, Hospital/ Clinic, Government, Private organization, I don't know,
Other ____________________

Q140 In the long-run which country would you like to work in?
Options: In the country where I currently work, In another EU country, In a non-EU country

Q141 You have reached the end of the survey. Do you have any final remarks?
____

The answers you have provided are most strictly confidential. After the data collection, all direct personal identifying information (e.g., name, date of birth) will be removed from all data. Our data protection statement contains more information on data protection and confidentiality.

Please reconfirm that you have provided consent for the use of your data for ERC evaluative purposes by checking YES below.

Do you consent to the processing of your personal information for the purposes of this study? Such information will be treated as strictly confidential and handled in accordance with the Regulation (EC) No 45/2001.

- ❍ Yes
- ❍ No

b) AdG survey

Welcome to the online ERCAREER inventory. Thank you very much for your interest in this research. We kindly remind you that you have previously provided consent for use of your application data for evaluative purposes to the ERC executive agency, which is why you are included in this study. ERCAREER's objective is to formulate specific and concrete recommendations for the development and improvement of the ERC's evidence-based policies, procedures, and practices to achieve greater gender balance among ERC applicants and grantees in particular, thereby contributing to the strive for gender equality in the European Research Area in general. If you have any questions or require more information about this study, please contact the researchers at: project.ercareer@gmail.com.

Additional notes

- Please use the arrow buttons to navigate through the inventory.
- Some questions are obligatory, in order to ensure the appropriate flow of the inventory and the most optimal results for our analyses.
- If you interrupt or have to stop the inventory and would like to continue at a later stage, simply close the screen. You can enter the inventory and resume at the same question at a later time by following the link in your email.

Section 1 - Your PhD (or equivalent doctoral degree)

We would like to start by asking a few questions about your PhD studies (or equivalent).

Q1 Which institution awarded your PhD (or equivalent)? Please write the full name of the institution / university.
Open text

Q2 In which country was the institution/university located?
[Dropdown menu] Countries

Q3 In what subject area was your PhD (or equivalent)?
Options:
Social Sciences and Humanities 1, Individuals, institutions and markets; Social Sciences and Humanities 2, Institutions, values, beliefs and behavior; Social Sciences and Humanities 3, Environment, space and population; Social Sciences and Humanities 4, The Human Mind and its complexity; Social Sciences and Humanities 5, Cultures and cultural production; Social Sciences and Humanities 6, The study of the human past; Social Sciences and Humanities ~ I don't know.
Physical Sciences and Engineering 1, Mathematics; Physical Sciences and Engineering 2, Fundamental constituents of matter; Physical Sciences and Engineering 3 Condensed matter physics; Physical Sciences and Engineering 4, Physical and analytical chemical sciences; Physical Sciences and Engineering 5, Synthetic chemistry and materials; Physical Sciences and Engineering 6, Computer science and informatics; Physical Sciences and Engineering 7, Systems and communication engineering; Physical Sciences and Engineering 8, Products and processes engineering; Physical Sciences and Engineering 9, Universe sciences; Physical Sciences and Engineering 10, Earth system science; Physical Sciences and Engineering ~ I don't know.
Life Sciences 1, Molecular and Structural Biology and Biochemistry; Life Sciences 2, Genetics, Genomics, Bioinformatics and Systems Biology; Life Sciences 3, Cellular and Developmental Biology; Life Sciences 4, Physiology, Pathophysiology and Endocrinology; Life Sciences 5, Neurosciences and neural disorders; Life Sciences 6 Immunity and infection; Life Sciences 7, Diagnostic tools, therapies and public health; Life Sciences 8, Evolutionary, population and environmental biology; Life Sciences 9, Applied life sciences and biotechnology; Life Sciences ~ I don't know

Q4 Did you have work experience of more than 6 months prior to your PhD? *Options:* Yes, No

If Yes Is Selected for Q4, If not skip to Q7

Q5 To what extent did this previous work experience help your PhD research?
Options: Not at all, A limited extent, Some extent, A significant extent, It was essential, Not applicable

Q6 To what extent did this previous work experience help your post-doctoral career?
Options: Not at all, A limited extent, Some extent, A significant extent, It was essential, Not applicable

Section 2 – Professional employment since your PhD (or equivalent doctoral degree)

In this section, we refer to "spells of employment". This refers to a period of employment during which both position and institution stay the same. This may include spells which started before you were awarded your PhD (or equivalent) but finished afterwards. The matrix below asks questions to describe your career history. Complete a row for each time you have SWITCHED position and/or institution since completing your PhD (or equivalent). If you worked at two institutions simultaneously, please complete a row for each employer. We will be asking you about any visiting or honorary positions or any leave taken later– so if any of these occurred whilst you had a contract, these should not be considered as separate spells. We will be asking you about any spells of unemployment or leave taken when you were not employed in Section 3.

We use the following classifications of the type of employment: **Post doc** - Research Assistant/Associate, Research Fellow, Researcher, Staff researcher; **Lecturer** - Scientist, Senior Researcher, (Junior) group/team leader, Assistant Professor, Lab head, Research Scientist, Principal Investigator, Staff scientist, Senior post-doctoral research fellow, Senior Research Scientist; **Senior lecturer** - Senior Scientist, Professor of Applied, Collaborative Researcher, Clinician Scientist, Chargé de recherche, Associate Professor, Habilitation, Permanent research scientist, Reader; **Professor** - Research Director, Full Professor, Directeur de recherché; **Other** - Consultant, Engineer, Special Researcher, Research Professor

Please fill out your spells of employment, both full and part-time, starting with your first position after your PhD (or equivalent). This may include spells which started before you were awarded your PhD but finished afterwards. Spells refer to any paid positions, regardless of hours (full vs part time) or contract status (permanent vs fixed term). For your current position, please write the current month and current year as the End date.

Spell	Q7 Type of employment	Q8 Employment dates		Q9 Institution type	Q10 Institution	Q11 Country
1-20	*Options:* Postdoc, Lecturer, Senior Lecturer, Professor, Other	Start MM/YYYY	End MM/YYYY	*Options:* Higher Education/ University, Not for profit research organization, Commercial research organization, Hospital/ Clinic, Government, Private organization, Other	Open text	Open text

Q12 If any of your responses to institution / organization type was "Other", please provide details on the type of industry where you spent most of this time, e.g., banking, consulting, manufacturing, self-employment. ______________________

Q13 When did you become an independent researcher? (undertaking research or leading a research group without being supervised) If applicable, please specify the date.
[Dropdown menu] MM/YYYY

Q14 When were you appointed to your first permanent post? If applicable, please specify the date.
[Dropdown menu] MM/YYYY

Q15 When did you achieve tenure? If applicable, please specify the date.
[Dropdown menu] MM/YYYY

Q16 Are you able to identify a point in your career where you feel that your research career really 'took' off or gained momentum? If so, please specify the year.
[Dropdown menu] MM/YYYY

Q17 Have you ever held unpaid / honorary / visiting appointments? [Multiple answers possible]
Options: No; Yes, honorary appointment; Yes, visiting appointment;
Yes, other ____________________

Q18 Were any of the unpaid / honorary / visiting appointments particularly useful for your career? Please explain in what respect this appointment / these appointments helped your career.

____

Q19 If you have changed institutions or geographic location during your career, why did you decide to move? [Multiple answers possible]
Options: Not applicable, End of contract, Promotion, Better facilities, Better institution, Better contract, Return to country of birth, Partner's career, Other ____________________

Q20 Have there been times when you have considered moving (e.g., to a different country or another institution) but did not move?
Options: Yes, No

Q21 Why did you decide not to move? [Multiple answers possible]
Options: Excellence of existing institution; Excellence of existing research group; Family or other responsibilities; Consequence of partner's career; Other ____________________

Q22 What is the number of hours per week specified in your current contract (if applicable)?
Open text

Q23 How many hours do you actually work per week?
Open text

Q24 Thinking about your career, have you usually worked:
Options: The same hours, More hours, It has varied, depending on the work at the time, Fewer hours

Q25 How many PhDs have you supervised to completion during your career?
Open text

We would like to learn more about the activities you are/were involved in during your <u>first position</u> after your PhD. Please make sure the percentages add up to a 100.

Q26 Main duties	Q27 Time involved in research	Q28 Involved in teaching?	Q29 Time involved in teaching	Q30 Involved in administration or management tasks	Q31 Time involved in administration or management	Q32 Involved in other activities (e.g. clinical responsibilities)	Q33 Time involved in other activities
Options: Research, Not research	%	*Options:* Yes, No	%	*Options:* Yes, No	%	*Options:* Yes, No	%

Q34 What other activities were you involved in during your first position after your PhD?

____

We would like to learn more about the activities you are/were involved in during your <u>first permanent academic position</u>. Please make sure the percentages add up to a 100.

Q35 Main duties	Q36 Time involved in research	Q37 Involved in teaching?	Q38 Time involved in teaching	Q39 Involved in administration or management tasks	Q40 Time involved in administration or management	Q41 Involved in other activities (e.g. clinical responsibilities)	Q42 Time involved in other activities
Options: Research, Not research	%	*Options:* Yes, No	%	*Options:* Yes, No	%	*Options:* Yes, No	%

Q43 What other activities were you involved in during your first permanent academic position after your PhD? _______________

We would like to learn more about the activities you are involved in during your current position. Please make sure the percentages add up to a 100.

Q44 Main duties	Q45 Time involved in research	Q46 Involved in teaching?	Q47 Time involved in teaching	Q48 Involved in administration or management tasks	Q49 Time involved in administration or management	Q50 Involved in other activities (e.g. clinical responsibilities)	Q51 Time involved in other activities
Options: Research, Not research	%	*Options:* Yes, No	%	*Options:* Yes, No	%	*Options:* Yes, No	%

Q52 What other activities were you involved in during your first permanent academic position after your PhD? _______________

Section 3 –Part time employment and Leave

Q53 Did you ever have a contract that was less than full time?
Options: Yes, No

If Yes Is Selected for Q53, if No skip to Q57

Please fill out one row for every period of part time employment. (Note: this question does not refer to periods of leave which will be covered separately).

Spell	Q54 Dates of Part-time employment		Q55 How many FTE did you work?	Q56 Reasons for Part-time employment
1-20	Start MM/YYYY	End MM/YYYY	Open text	*Options:* Family responsibilities, Further study, Health, Only work available, Pursuing other interests, Quality of life, Research project (externally funded), Other

Q57 Have there been (other) times when you have considered but not requested part time employment?
Options: Yes, No

Q58 Why did you decide not to request part time employment? _______________________

Q59 Did you take any leave during your career? (e.g., research leave, parental leave, military leave, medical training, leave due to illness, etc)
Options: Yes, No

If Yes Is Selected for Q59, if No skip to Q65

Please fill out one row for every different type of leave you took (this can also be multiple leaves of the same type at different times), even if they occurred at the same time.

Spell	Q60 Type of leave	Q61 Dates of leave		Q62 Did you take paid or unpaid leave?	Q63 Did you take full-time or part-time leave?
1-20	*Options:* Research leave, Parental leave (incl. maternity leave, paternity leave), Military leave, Medical training, Leave due to long-term illness, Other	Start MM/YY YY	End MM/YY YY	*Options:* Paid, Unpaid	*Options:* Full-time, Part-time

Q64 If any of your responses to type of leave was 'Other leave', please provide details on the type of leave you have taken. ____

Q65 Have there been (other) times when you have considered but not taken leave?
Options: Yes, No

Q66 Why did you decide not to take leave? ________________

We would like to know whether there is a formal institutional or national system / mechanism which takes account of "time to care" in promotion or tenure decisions, performance measurement or in research time allocation. [Select all that apply]

Q67 **Extension of window**. Stopping the clock for tenure and promotion decisions (e.g. 12 months per child or actual time of leave taken into account)
Q68 **Adaption of criteria**. Compensation for part-time work (e.g. working 80% or 0.8 FTE implies criteria for promotion of research time allocation are set at 80%)
Q69 **Compensation for time to care** (e.g. temporary reduced teaching load to invest in research productivity upon return)

Options: I am not aware of this support, I am aware that this exists, This is available in my institution, I have used this.

Have any of the following working conditions ever been influential in your choice of employer?

Q70 Availability of reduced hours (part time)
Q71 Availability of annualised hours (working fixed number of hours a year, flexibility in when to work)
Q72 Availability of compressed hours (e.g. 4 * 9 contract)
Q73 Availability of flexi-time/ flexible hours
Q74 Availability of term time (during the school year) working
Q75 Availability of another type of flexible contract

Options: Never been important, became important after I had children, always been important

Section 4 - Spells outside of employment

We are now interested in times when you did not have an employment contract (if applicable).

Q76 Since completing your PhD (or equivalent), have there been times when you did not have an employment contract (through unemployment, unpaid full time leave, or retirement)?
Options: Yes, No

Q77 Are you currently without an employment contract?
Options: Yes, No

Q85 Are you a parent?
Options: Yes, No

If Yes Is Selected for Q85, if No skip to Q88

Q86 How many children (biological / adopted / step children) do you have?
Options: 1-10, >10

Q87 Can you please give the date(s) of birth?
[Drop down menu for each child] MM/YYYY

To what extent do / did you have care responsibilities?

	Care responsibilities *Options:* Yes, No	During what stage of your career? *Options:* Early, Mid, Current	Share in care responsibilities *Options:* Sole, Main (>50% but < 100%), Equal, Shared (>)% but less than 50%)
Q88 Children			
Q89 Elderly parents			
Q90 Partner			
Q91 Other family members/dependents			

Q92 At present, do you have a spouse or partner?
Options: Yes, No

If Yes Is Selected for Q92, if No skip to Q104

Q93 Since what year have you and your spouse or partner been together?
[Drop-down menu] (YYYY)

Q94 Do you and your spouse or partner live in the same household?
Options: Yes, all of the time, Yes, most of the time, No

Q95 What is the highest formal qualification of your spouse or partner?
Options: Doctorate/DPhil/PhD, MBA, Professional Law/ Financial/ Business qualification, Masters degree, Bachelors degree, Secondary education, Other ____________________

Q96 Is your spouse or partner employed?
Options: Yes, in full time employment; Yes, in part time employment; No, in education; No, taking leave; No, full time care responsibilities; No, retired; No, other ____________________

If Yes Is Selected for Q96, if No skip to Q101

Q97 Is your spouse or partner also a scientist/researcher/academic?
Options: Yes, No

Q98 Is your partner working in a similar institution?
Options: Yes, No

Q99 Is your spouse or partner working in the same institution?
Options: Yes, No

Q100 How many hours per week does your spouse or partner work?
Open text

Q101 How easy has it been over the years to combine dual careers?
Options: Very difficult, Difficult, Neither easy nor difficult, Easy, Very easy

Q102 During your relationship, whose career has been more important?
Options: Mine, Mostly mine, Both equally, Mostly my spouse or partner's career, My spouse or partner's career, Not applicable

Q103 Reflecting back over time, if you say your careers were more or less equally important, would you qualify the development process of your careers as …
Options: Synchronic (careers developed in a similar way at a similar time), Competing (one career developed at the expense of the other or vice versa), Independent (careers developed independently of each other)

Q104 People differ in their perception of the non-work conditions for a successful career in science. Please indicate to what extent you agree with the following statement. For a successful research career, you need a spouse or partner who takes care of the family and household.
Options: Strongly disagree, Disagree, Neither agree nor disagree, Agree, Strongly agree

Section 7 - Applying to the ERC for AdG funding

In this section, we are interested to know more about you ERC application.

Q105 Have you applied to other funding agencies for support with this research? [Select all that apply]
Options: No, only to the ERC; Yes, to other institutional research funding programs; Yes, to other national research funding programs; Yes, to other international research funding programs;
Yes, other ____________________

Q106 Have you made other applications to the ERC? [Multiple answers possible]
Options: No; Yes, AdG 2008; Yes, AdG 2009; Yes, AdG 2010; Yes, AdG 2011; Yes, AdG 2012; Yes, AdG 2013; Yes, other ____________________

Q107 In making your most recent ERC application did you receive support from within your institution?
Options: Not at all, Limited, Occasional, Extensive, All

Q108 What type of support did you receive? [Select all that apply]
Options: Formal support - e.g., Institution requires an internal peer review before submission; Informal support - e.g., You approached a colleague to read through your application and provide feedback; Other ____________________

Q109 In making your most recent ERC application did you receive support from outside your institution?
Options: Not at all, Limited, Occasional, Extensive, All

Q110 What type of support did you receive? [Select all that apply]
Options: Formal support - e.g., Institution requires an internal peer review before submission; Informal support - e.g., You approached a colleague to read through your application and provide feedback; Other ____________________

Q111 You have reached the end of the survey. Do you have any final remarks?
____

The answers you have provided are most strictly confidential. After the data collection, all direct personal identifying information (e.g., name, date of birth) will be removed from all data. Our data protection statement contains more information on data protection and confidentiality.

Please reconfirm that you have provided consent for the use of your data for ERC evaluative purposes by checking YES below.

Do you consent to the processing of your personal information for the purposes of this study? Such information will be treated as strictly confidential and handled in accordance with the Regulation (EC) No 45/2001.
❍ Yes
❍ No
