# Supplementary material for: Mapping career patterns in research: A sequence analysis of career histories of ERC applicants
Source: PLoS One. 2020 Jul 29;15(7):e0236252. doi: 10.1371/journal.pone.0236252 (PMC7390397; doi:10.1371/journal.pone.0236252)
Supplement: S3 File — (DOCX) [file pone.0236252.s003.docx]

**Supplementary Materials**

**SM 1) Notes**

**ERC funding schemes**

**SM 2) Analyses**

**Robustness tests**

**SM 3) Tables**

**ST.1) Samples benchmarked against the ERC population**

**ST.2) Categorization of job positions**

**ST.3) Descriptive statistics – domain, gender, success, parenthood, partnership, nationality, host institution**

**ST.4) Descriptive statistics – leave**

**ST.5) StG – gender-specific effects of PhD characteristics**

**ST.6) StG – gender-specific effects of PhD characteristics**

**SM 4) Figures**

**SF.1) Dendrograms for StG (SF.1a) and AdG (SF.1b) applicants**

**SM 1) Supplementary notes**

**ERC funding schemes**

The European Research Council (ERC) established the Starting and Advanced grant schemes in 2007, <http://erc.europa.eu/funding-and-grants/funding-schemes>. T**he Starting Grant scheme is intended for researchers up to 12 years after their PhD. In 2012 when we collected our data, applicants to the Starting Grant were sorted into either starters (within 7 years of a PhD) or consolidator (8-12 years after a PhD). Applicants had the possibility to change stage from consolidator to starter or to extend the consolidator window on account of maternity leave (18 months per child), parental leave, illness, or military service. The Advanced Grant is aimed at established researchers with a strong research record who would be considered to be leaders in their field.**

**Applications are accepted across disciplines and considered by expert sub-panels within the umbrella of three domains: LS – life sciences; PE – physical sciences and engineering; SH – social sciences and humanities.**

Life Sciences: Molecular and Structural Biology and Biochemistry; Genetics, Genomics, Bioinformatics and Systems Biology; Cellular and Developmental Biology; Physiology, Pathophysiology and Endocrinology; Neurosciences and neural disorders; Immunity and infection; Diagnostic tools, therapies and public health; Evolutionary, population and environmental biology; Applied life sciences and biotechnology.

Physical Sciences and Engineering: Mathematics; Fundamental constituents of matter; Condensed matter physics; Physical and analytical chemical sciences; Synthetic chemistry and materials; Computer science and informatics; Systems and communication engineering; Products and processes engineering; Universe sciences; Earth system science.

Social Sciences and Humanities: Individuals, institutions and markets; Institutions, values, beliefs and behavior; Environment, space and population; The Human Mind and its complexity; Cultures and cultural production; The study of the human past.

**SM 2) Supplementary analyses**

**Robustness tests**

We tested the consistency of the career patterns by estimating the OMA and cluster analysis for three different specifications. Further details are available from the authors upon request.

First, we re-estimated the OMA and cluster analysis for the StG sample by making our period of analysis consistent with that used for the ADG sample, excluding the period between the PhD and first job, starting the period of observation with the date of the first job position. Applying a five-cluster solution, the analysis confirms the distinct patterns. There are slight variations in the size of the clusters. The relative shares of steady progress at universities and in research institutes decline, the other three clusters gain in relative importance. The results of the multinomial logit lead to similar conclusions.

Second, we re-estimated the OMA and cluster analysis for the AdG sample reducing the period of observation for all individuals in the sample to a maximum of 300 months, thereby reducing the influence of the very long careers of some scientists in our sample. Applying a five-cluster solution, the analysis suggests that the clusters of steady progress in research institutes and steady progress in government are robust. The pattern of complicated moves across institutions gains in relative importance. The analysis also confirms that there are two distinct clusters in universities, but the cluster sizes are much more equal. The smaller cluster comprises scientists with on average longer careers and who have made quicker career progression across job positions than the scientists in the other steady progress in universities cluster. We also found this difference in the analysis presented in the main text. The results of the multinomial logistic regressions are robust, but show some more distinct predictors for this quicker career cluster. Female scientists, for example, and scientists from younger birth cohorts are still less likely in this cluster.

Third, we conducted OMA and cluster analysis for a pooled StG and AdG sample. Focusing on early careers, we differentiated between seven years after PhD, as this is the formal cut-off point for a “starter” in the application process, nine years as this reflects the average career duration of our actual StG sample, and twelve years to reflect “consolidators”. Regardless of whether we look at the first 7, 9 or 12 years of the careers for the pooled sample, we still identify a pattern of *complex moves across institutions*, as well as several distinct career patterns within universities which seem to reflect the speed at which promotions occur. The presence of a distinct career pattern in research institutes becomes more apparent when we analyze longer career spells - the first nine or twelve years of the careers.

**SM 3) Supplementary Tables**

***ST.1) Samples benchmarked against the ERC population***

|  | Population | Consent sample | Survey sample | |
| --- | --- | --- | --- | --- |
|  |  |  |  | % of consent sample |
| *StG 2012* | 4741 | 1588 | 339 | 21.3 |
| Grantees (%) | 11.7 | 11.5 | 17.4 |  |
| Women (%) | 28.9 | 29 | 39.2 |  |
| LS (%) | 34.9 | 36 | 38.3 |  |
| PE (%) | 43.4 | 42.8 | 41.6 |  |
| SH (%) | 21.7 | 21.2 | 20.1 |  |
| *AdG* | 10348 | 4088 | 746 | 18.2 |
| Grantees (%) | 13.5 | 19.7 | 21.4 |  |
| Women (%) | 14.6 | 15.4 | 19.6 |  |
| LS (%) | 33.5 | 33.9 | 30.8 |  |
| PE (%) | 43.8 | 41.8 | 45.4 |  |
| SH (%) | 22.8 | 24.3 | 23.7 |  |

LS=Life Sciences, PE= Physical Sciences and Engineering, SH = Social Sciences and Humanities

***ST.2) Categorization of job positions***

| Position labels | Includes comparable job descriptions from different national and discipline-specific contexts, based on ERCAREER CV coding scheme | EU/MORE | SheFigures | LERU |
| --- | --- | --- | --- | --- |
| Post doc | Post doctorate, Research Assistant/Associate, Research Fellow, Researcher, Staff researcher, LERU Phase 2 | R2 | - | 2 |
| Lecturer | Lecturer, Scientist, Senior Researcher, (Junior) group/team leader, Assistant Professor, Lab head, Research Scientist, Principal Investigator, Staff scientist, Senior post-doc research fellow, Senior Research Scientist, LERU Phase 3a | R2/3 | C | 3a |
| Senior lecturer | Senior lecturer, Senior Scientist, Collaborative Researcher, Clinician Scientist, Chargé de recherche, Associate Professor, Habilitation, Permanent research scientist, Reader, LERU Phase 3b | R3 | B | 3b |
| Professor | Professor, Research Director, Full Professor, Directeur de recherche, LERU Phase 4 | R4 | A | 4 |
| Other | e.g. Special Researcher, Research Professor |  |  |  |

Sources:

League of European Research Universities (LERU) framework 2010

<http://www.leru.org/files/publications/LERU_paper_Harvesting_talent.pdf>

SheFigures 2012

<http://ec.europa.eu/research/science-society/document_library/pdf_06/she-figures-2012_en.pdf>

EU framework = MORE

<https://cdn5.euraxess.org/sites/default/files/policy_library/towards_a_european_framework_for_research_careers_final.pdf>

***ST.3) Descriptive statistics of survey samples***

|  | ***StG*** | | | | ***AdG*** | | | |
| --- | --- | --- | --- | --- | --- | --- | --- | --- |
|  | ***%/***  ***Mean*** | ***St Dev*** | ***Min*** | ***Max*** | ***%/***  ***Mean*** | ***St Dev*** | ***Min*** | ***Max*** |
| *Discipline* |  |  |  |  |  |  |  |  |
| Life sciences | 0.38 |  |  |  | 0.31 |  |  |  |
| Physical sciences & engineering | 0.42 |  |  |  | 0.45 |  |  |  |
| Social science & humanities | 0.20 |  |  |  | 0.24 |  |  |  |
| *PhD related characteristics* | | |  |  |  |  |  |  |
| Age^+^ | 31.04 | 3.85 | 23 | 56 | 28.96 | 4.59 | 21 | 52 |
| Age missing | 0.02 |  |  |  | 0.01 |  |  |  |
| PhD in home country | 0.78 |  |  |  | 0.79 |  |  |  |
| *Leiden score of PhD Institution* | | |  |  |  |  |  |  |
| PP | 11.66 | 3.06 | 4.4 | 23 | 12.49 | 3.74 | 3.9 | 25.2 |
| Leiden score missing | 0.18 |  |  |  | 0.15 |  |  |  |
| *Children^+^ (ref.: no children)* | 0.15 |  |  |  | 0.29 |  |  |  |
| Age of youngest child^+^ | |  |  |  |  |  |  |  |
| No children | 0.85 |  |  |  | 0.72 |  |  |  |
| Youngest child ≤3 | 0.08 |  |  |  | 0.18 |  |  |  |
| Youngest child > 3 | 0.07 |  |  |  | 0.11 |  |  |  |
| *Work experience before PhD* | | |  |  |  |  |  |  |
| None | 0.50 |  |  |  | 0.45 |  |  |  |
| *Personal characteristics* | |  |  |  |  |  |  |  |
| Female (ref.: Male) | 0.39 |  |  |  | 0.20 |  |  |  |
| *Nationality* |  |  |  |  |  |  |  |  |
| Central Europe | 0.38 |  |  |  | 0.49 |  |  |  |
| East Europe | 0.10 |  |  |  | 0.09 |  |  |  |
| Southern Europe | 0.34 |  |  |  | 0.27 |  |  |  |
| Northern Europe | 0.07 |  |  |  | 0.07 |  |  |  |
| Rest of world | 0.10 |  |  |  | 0.07 |  |  |  |
| *N* | 322 |  |  |  | 737 |  |  |  |

^+^StG: when received PhD, AdG: when started first job after PhD

***ST.4) Researchers with leave in career (%)***

|  | ***All*** | ***Men*** | ***Women*** | ***Non-Grantees*** | ***Grantees*** |
| --- | --- | --- | --- | --- | --- |
| *StG (n=339)* |  |  |  |  |  |
| Any leave | 34 | 25 | 47 | 35 | 29 |
| Parental leave | 22 | 11 | 40 | 22 | 22 |
| *AdG (n=745)* |  |  |  |  |  |
| Any leave | 46 | 41 | 70 | 46 | 48 |
| Parental leave | 45 | 40 | 69 | 45 | 48 |

***ST.5 StG – gender-specific effects of PhD characteristics (Average Marginal Effects [AME] from multinomial logistic regressions with interaction terms, 95% confidence intervals [CI])***

|  | ***steady progress*** | | | | ***quick advances*** | | | | ***delayed advances*** | | | | ***steady progress*** | | | | ***complicated moves*** | | | |
| --- | --- | --- | --- | --- | --- | --- | --- | --- | --- | --- | --- | --- | --- | --- | --- | --- | --- | --- | --- | --- |
|  | ***in universities*** | | | | ***in universities*** | | | | ***in universities*** | | | | ***in research institutes*** | | | | ***across institutions*** | | | |
|  | AME |  | 95% CI | | AME |  | 95% CI | | AME |  | 95% CI | | AME |  | 95% CI | | AME |  | 95% CI | |
| *PhD in home country (Ref, Int, Mobile for PhD)* | | | | | |  |  |  |  |  |  |  |  |  |  |  |  |  |  |  |
| Male | .049 |  | -.101 | .199 | -.048 |  | -.186 | .091 | .039 |  | -.104 | .183 | .039 |  | -.104 | .183 | -.020 |  | -.143 | .101 |
| Female | .144 | + | -.017 | .305 | -.043 |  | -.056 | .071 | -.088 |  | -.278 | .102 | -.088 |  | -.278 | .103 | .032 |  | -.141 | .205 |
| *Leiden score of PhD Institution* | | | |  |  |  |  |  |  |  |  |  |  |  |  |  |  |  |  |  |
| Male | -.001 |  | -.023 | .020 | .011 |  | -.007 | .029 | .018 | + | -.003 | .039 | .008 |  | -.008 | .024 | -.035 | ** | -.059 | -.012 |
| Female | -.004 |  | -.030 | .023 | -.004 |  | -.022 | .013 | -.001 |  | -.027 | .026 | .008 |  | -.018 | .032 | .001 |  | -.028 | .028 |
| *Age when received PhD* | | |  |  |  |  |  |  |  |  |  |  |  |  |  |  |  |  |  |  |
| Male | .003 |  | -.022 | .028 | -.022 | * | -.043 | -.001 | -.010 |  | -.035 | .015 | .008 |  | -.006 | .023 | .021 | * | .003 | .040 |
| Female | .001 |  | -.024 | .026 | -.016 | + | -.034 | .001 | .009 |  | -.015 | .034 | -.018 |  | -.046 | .010 | .024 | * | .001 | .047 |
| *Age of youngest kid when received PhD (Ref,: no kids)* | | | | | |  |  |  |  |  |  |  |  |  |  |  |  |  |  |  |
| < 3 |  |  |  |  |  |  |  |  |  |  |  |  |  |  |  |  |  |  |  |  |
| Male | -.056 |  | -.245 | .132 | .150 |  | -.056 | .358 | -.062 |  | -.231 | .106 | .045 |  | -.102 | .193 | -.077 |  | -.236 | .082 |
| Female | -.287 | *** | -.372 | -.203 | .112 |  | -.203 | .427 | .165 |  | -.346 | .476 | .085 |  | -.369 | .541 | .024 |  | -.429 | .478 |
| >=3 |  |  |  |  |  |  |  |  |  |  |  |  |  |  |  |  |  |  |  |  |
| Male | -.283 | *** | -.351 | -.214 | .164 |  | -.149 | .478 | -.073 |  | -.379 | .232 | .166 |  | -.186 | .517 | .025 |  | -.258 | .310 |
| Female | -.255 | *** | -.364 | -.148 | -.029 |  | -.114 | .056 | .530 | ** | .226 | .834 | -.061 |  | -.294 | .171 | -.183 | * | -.340 | -.026 |

Sig.: *** p<0.001, ** p<0.01, * p<0.05, + p<=0.1

Note: Results estimated from separate multinomial logistic regressions for each interaction terms between gender and personal and PhD-related characteristic including all other variables.

Legend: For categorical variables, the AME indicate by how many percentage points the probability of being in a certain pattern is on average higher or lower for a scientist with certain personal and PhD characteristics compared to the reference category. For metric variables, such as age and Leiden score, the AME indicate by how many percentage points the probability of being in a certain cluster increases (decreases) if the variable increases (decreases) by one unit. Differences in the effects between bale and Female researchers are statistically significant if the estimated confidence intervals do not overlap.

***ST.6 AdG – gender-specific effects of PhD characteristics (Average Marginal Effects [AME] from multinomial logistic regressions with interaction terms, 95% confidence intervals [CI])***

|  | ***steady progress*** | | | | ***steady progress, matured*** | | | | ***steady progress*** | | | | ***steady progress*** | | | | ***complicated moves*** | | | |
| --- | --- | --- | --- | --- | --- | --- | --- | --- | --- | --- | --- | --- | --- | --- | --- | --- | --- | --- | --- | --- |
|  | ***in universities*** | | | | ***in universities*** | | | | ***in research institutes*** | | | | ***at government institutes*** | | | | ***across institutions*** | | | |
|  | AME |  | 95% CI | | AME |  | 95% CI | | AME |  | 95% CI | | AME |  | 95% CI | | AME |  | 95% CI | |
| *PhD in home country (Ref. Int. Mobile for PhD)* | | | | | | | |  |  |  |  |  |  |  |  |  |  |  |  |  |
| Male | -.040 |  | -.138 | .058 | .009 |  | -.064 | .083 | .039 |  | -.016 | .095 | .002 |  | -.041 | .046 | -.010 |  | -.067 | .047 |
| Female | -.039 |  | -.241 | .163 | .095 |  | -.021 | .211 | -.032 |  | -.193 | .127 | .005 |  | -.117 | .128 | -.029 |  | -.152 | .094 |
| *Leiden score PhD Institution* | | | |  |  |  |  |  |  |  |  |  |  |  |  |  |  |  |  |  |
| Male | .004 |  | -.008 | .015 | .001 |  | -.007 | .009 | .002 |  | -.005 | .010 | -.003 |  | -.009 | .003 | -.003 |  | -.010 | .003 |
| Female | .015 |  | -.009 | .039 | .003 |  | -.014 | .019 | -.007 |  | -.025 | .011 | -.021 | * | -.040 | -.001 | .010 |  | -.003 | .024 |
| *Age when received PhD* | | | |  |  |  |  |  |  |  |  |  |  |  |  |  |  |  |  |  |
| Male | .017 | *** | .008 | .027 | -.019 | *** | -.026 | -.012 | -.001 |  | -.009 | .005 | -.002 |  | -.006 | .003 | .005 | * | .000 | .010 |
| Female | .021 | ** | .006 | .037 | -.018 | ** | -.029 | -.006 | .001 |  | -.011 | .013 | -.008 | + | -.018 | .001 | .003 |  | -.006 | .012 |
| *Age of youngest kid in 1st job after PhD (Ref.: no kids)* | | | | | | | |  |  |  |  |  |  |  |  |  |  |  |  |  |
| < 3 |  |  |  |  |  |  |  |  |  |  |  |  |  |  |  |  |  |  |  |  |
| Male | -.045 |  | -.146 | .055 | .010 |  | -.061 | .082 | -.001 |  | -.069 | .069 | -.022 |  | -.060 | .018 | .056 |  | -.010 | .123 |
| Female | -.061 |  | -.276 | .155 | .114 |  | -.049 | .278 | -.045 |  | -.191 | .102 | .038 |  | -.108 | .185 | -.047 |  | -.159 | .062 |
| >=3 | |  |  |  |  |  |  |  |  |  |  |  |  |  |  |  |  |  |  |  |
| Male | .060 |  | -.071 | .192 | -.078 |  | -.172 | .016 | -.037 |  | -.113 | .038 | .015 |  | -.059 | .088 | .041 |  | -.035 | .117 |
| Female | .010 |  | -.220 | .241 | .133 |  | -.072 | .340 | -.049 |  | -.200 | .101 | -.066 | ** | -.115 | -.018 | -.028 |  | -.142 | .085 |

Sig.: *** p<0.001, ** p<0.01, * p<0.05, + p<=0.1

Note: Results estimated from separate multinomial logistic regressions for each interaction terms between gender and personal and PhD-related characteristic including all other variables.

Legend: For categorical variables, the AME indicate by how many percentage points the probability of being in a certain pattern is on average higher or lower for a scientist with certain personal and PhD characteristics compared to the reference category. For metric variables, such as age and Leiden score, the AME indicate by how many percentage points the probability of being in a certain cluster increases (decreases) if the variable increases (decreases) by one unit. Differences in the effects between male and female researchers are statistically significant if the estimated confidence intervals do not overlap.

**Supplementary Figures SF.1-SF.3**

***SF.1) Dendrograms***

SF.1a) StG applicants

SF.1b) AdG applicants
