# Supplementary material for: Mapping career patterns in research: A sequence analysis of career histories of ERC applicants
Source: PLoS One. 2020 Jul 29;15(7):e0236252. doi: 10.1371/journal.pone.0236252 (PMC7390397; doi:10.1371/journal.pone.0236252)
Supplement: Attachment — Submitted filename: Response to Reviewers 1.docx [file pone.0236252.s004.docx]

Amsterdam, April 17, 2020

Editorial board, *PLOS ONE*

Dear editor:

With this letter, we submit our revised manuscript “Mapping career patterns in research: A sequence analysis of career histories of ERC applicants” [PONE-D-19-29861]

to *PLOS ONE.* Thank you also for extending our deadline.

We thank the editor and the two reviewers for their kind words and insightful suggestions, which we hope to have addressed in this revision.

The earlier edition of the literature review of career patterns in research described very briefly in the previous version of the manuscript (find details of all studies included in the review in the supplementary materials) has now been published and the reference to this chapter is incorporated in the manuscript.

We have prepared high resolution graphic files for the Figures and tested these using the "preflight" PACE tool. We now also include the survey questions in the supplementary materials.

Unrestricted and uncontrolled access to the complete career history data (in terms of position, institution, contract type, location etc. of all spells since PhD) compromises the confidentiality and privacy of research participants, and violates the conditions on ethics approval obtained for this study from the Ethical Committee of the European Research Council. A replication of the optimal matching analysis is therefore not possible.

To meet the *PLOS ONE* data access policy, however, we are in the process of preparing simplified de-identified data files (containing relevant personal and career related variables used in the multinomial regression analyses, including a career pattern denominator) and have requested permission from the ERC for open or limited access to these files. We will follow up with the editor once we hear back from the ERC.

We look forward to hearing from you soon.

With kind regards,

Claartje Vinkenburg, Sara Connolly, Stefan Fuchs, Channah Herschberg, and Brigitte Schels

*Comments to the author:*

*2. Please include additional information regarding the survey or questionnaire used in the study and ensure that you have provided sufficient details that others could replicate the analyses.*

The pdf versions of the survey for both samples have now been provided in the supplementary materials

*3. We note that you have indicated that data from this study are available upon request. PLOS only allows data to be available upon request if there are legal or ethical restrictions on sharing data publicly.*

See above

*4. Please include your tables as part of your main manuscript and remove the individual files. Please note that supplementary tables (should remain/ be uploaded) as separate "supporting information" files*

Tables now included in main manuscript

*5. Review Comments to the Author*

*Reviewer #1: This is a review of Mapping Career Patterns in Research: A Sequence Analysis of Career Histories of ERC Applicants.*

*This paper is straightforward, clear, reproducible (provided that the authors' offer to provide survey data to those interested), and interesting. The authors take careers trajectories, as reported by ERC applicants on a survey, and map them to sequences. The sequences are then compared pairwise and clustered into canonical career trajectories, making it then straightforward to ask questions like: are there differences in the demographic makeup of sequence clusters, or, do applicants from a particular cluster tend to get funded more often than others.*

*Below, I have some brief suggestions for clarity, but these are merely suggestions. The paper, as it stands, is recommended for publication. I'd like to thank the authors for writing something so clearly without attempting to overstate or understate their conclusions. It was a pleasure to read and review.*

Thank you for your kind words and compliments. Much appreciated!

*My only question is whether the survey data's limited availability complies with the PLOS data access policy. I leave it to the editor and authors to decide that.*

See response to Editor above

*Suggestions: 1. After saying "process outcomes" in quotation marks, it would be good to explain to the reader what this means.*

We have changed this line on p2 to reflect that while others look at attainment, publications or prestige as career outcomes, we follow Abbot (2005) in viewing the pattern *itself* as an outcome

*2. Why are there {reference withheld} indications, and will they be included in the published version?*

See response to Editor above, this book chapter has now been published so reference is included

*3. "Since 2013, the Starting and Consolidator are separate grant schemes but at the time we collected our data, this was still one call." I'm not sure what "this was still one call" means. Can this be stated more clearly?*

We have changed this statement on p9 to reflect the fact that at the time that our data was collected there was a single Starter grant scheme which was open to starters (<7 years after a PhD) and consolidators (8-12 years after a PhD).

*4. "this project was commissioned by the ERC gender balance thematic working group to explore gender aspects in career structures and career paths of applicants." I wouldn't mind knowing this purpose earlier. It places the access to data and reason for the study in context.*

We have moved this statement to earlier in the paper, now on p3

*5. Can the figures be made larger or rearranged somehow? Even zooming in, it was hard to read them, but they are important to the findings of the paper, and are presented / formatted nicely. Given that there are no other figures in the paper, I'd suggest taking up a bit more space!*

High resolution files have been tested using PACE and uploaded

*6. For figure captions, the non-abbreviated StG and AdG might be nice so that the skimming reader, problematic though such a reader may be, can dip in and still understand these punchline figures.*

This has been changed as suggested

*7. The paragraph on pages 14-15 that describes the demographic differences between the career patterns by success rate and gender lead with differences, but then state that those differences are not significant. This statement of "there are differences but they are not actually differences" is a little confusing, and I would suggest, if the authors feel comfortable, changing wording so that it is clear that it "appears at first glance" that there are differences or something like that, so that the language better reflects the statistical conclusions.*

We have clarified the language on P15-16, as well as the language on the weighting on P11, to reflect this

*8. "linked to the high proportion of LS in research institutes"*

*By the time I got to this point, I had sort of lost the thread on the Life Sciences abbreviation. No need to make the decision based solely on my comments, but do the acronyms buy that much space saving or clarity?*

We have made sure that we refer to "Life Sciences" (not LS) at the beginning of each new section and where relevant

*9. Overall I thought that the references were appropriate and thorough. Nothing to add.*

*Trivial changes or typos*	thank you, these have been corrected

*Reviewer #2: The paper "Mapping Career Patterns in Research: A sequence Analysis of Career Histories of ERC Applicants" by Vinkenburg, Connolly, Fuchs, Herschberg and Schels presents a survey-based analysis, categorizing career patterns by their progression through career stages (sequences) and locations. They follow up the categorization with an analysis of the parameters describing who follows which pattern.*

*The study is innovative in the way data and analysis is combined, and it is a highly relevant topic. I applaud the authors for a well-planned research design.*

Thank you for your kind words and compliments.

*I would be happy to recommend this paper for acceptance, but have a few minor comments, which the authors might consider as possible improvements to their paper. I also need to know why some of the references are withheld - it is not clear from the context why this is the case, or if they will be included at the time of publication.*

This chapter has now been published and the reference is included

*Suggested revisions: I appreciate the review section. However, it is at times a little bit superfluous in that it summarizes a number of studies very briefly without explicitly using that information for anything. It almost gives this reader the impression that those references are included more "for show". This is not something I want to think of an otherwise great paper, so I would suggest that the authors carefully consider if a) all references in the review are important to include and b) if they could add something more conclusive or argumentative to the more summarizing parts of the review.*

We have reworked and re-ordered the discussion of the literature review. The brief summary of the empirical studies on career patterns in research has been removed, but we have kept the most important and surprising finding (out of 40 found, there are only three (!) studies that empirically establish patterns) and discussed the two dominant themes. The total number of references in this section has been reduced and readers interested in details are directed to the supplementary materials in which the full review table is provided, as well as the book chapter in which an earlier version of this review is described.

*I also suggest expanding a little on the methods, especially OMA. It is good that the authors include references to previous uses and tests of OMA, but it would be great to see a slightly more technical explanation of the method, e.g. in terms of how observations are coded and compared, or alternatively an example of the matching using made-up data, that allows the reader to more clearly understand the procedure. Ward clustering is also described very briefly, but as this is a somewhat more well-known approach, I find that more acceptable. But the authors could consider expanding this section slightly as well, perhaps using a visual explanation.*

We have introduced an illustrative example to explain the OMA method, following [1]. Considering overall length and recognizing that Ward clustering is relatively well-known, we did not expand upon this. Indeed, the dendrograms provided in the supplementary materials offer a visualization of the clustering.

1.	Aisenbrey S, Fasang A. The Interplay of Work and Family Trajectories over the Life Course: Germany and the United States in Comparison. Am J Sociol. 2017;122: 1448–1484. doi:10.1086/691128
